# Genomic and expression analysis of a solute carrier protein (*CcSLC25a5*) gene from *Cyprinus carpio Linnaeus*

**DOI:** 10.1186/2193-1801-2-458

**Published:** 2013-09-12

**Authors:** Li Jiang, Anda Cheng, Yangyang Wang, Baoyong Zhang

**Affiliations:** The Center for Applied Aquatic Genomics, Chinese Academy of Fishery Sciences, Beijing, 100141 China; College of Fisheries and Life Science, Shanghai Ocean University, Shanghai, 201306 China; College of Fisheries and Life Science, Dalian Ocean University, Dalian, 116023 China

**Keywords:** *Cyprinus carpio Linnaeus*, Scale development, Solute carrier protein, Scale initiation, Embryo development

## Abstract

Using the Genefishing method, we identified seven potential regulatory genes involved in the process of scale morphogenesis in fishes. We further characterized a novel solute carrier protein gene (*CcSLC*)*,* from the common carp which is differentially expressed in mirror carp and Jianli. The ORF encodes a peptide of 298 amino acids with a molecular mass of 31.5 kDa and a theoretical isoelectric point of 7.49. ScanProsite analysis indicated that it is a putative solute carrier protein that contains a substrate binding site. *CcSLC* was detected in carp embryos by *in situ* hybridization in the 70%-epiboly, 6-somite, and 14-somite embryonic stages. Gene expression stopped at the long pec stage. However, *CcSLC25a5* was re-expressed during the initiation of scale formation in the regions that were scale covered. These findings provide novel insights into the features of early carp embryo and scale development.

## Introduction

Membrane transporters are the gatekeepers for all cells and organelles, controlling uptake and efflux of crucial compounds such as sugars, nucleotides, inorganic ions, and drugs (Hediger et al. 2004). They are responsible for substrate movement across both cytoplasmic membranes of cells and internal membranes of organelles (Sreedharan et al. 2011). Transporters can be divided into ABC transporters, pumps, ion channels, water channels, and solute carriers. Membrane bound proteins represent about 27% of the entire human proteome. Among the membrane bound proteins, the SLC transporters are the second largest group after G protein coupled receptors (Lagerstrom 2008 Almen et al. 2009). Transporters can be divided into two families, passive and active transporters. The active transporters use diverse energy-coupling mechanisms to allow the movement of molecules across a membrane against a concentration gradient. The passive transporters, also known as facilitated transporters, allow passage of solutes (e.g., glucose, amino acids, urea) across membranes down their electrochemical gradients (Hediger et al. 2004).

Appreciation of the role that transport proteins play in the absorption, distribution, and elimination of a wide variety of drugs in clinical use is increasing. As the largest group of secondary transporters, SLC transporters are becoming the focus of an increasing number of studies because they control transmembrane movement of many types of important substrates. The human genome contains approximately 360 unique SLC protein genes grouped into 48 families (Ren et al. 2007; Fredriksson et al. 2008). Approximately 19 of the SLC gene families have been reported to transport xenobiotics including: organic anion polypeptides (*SLCO*), oligopeptides (*SLC15*) (Russel et al. 2002; Brandsch et al. 2008; Dobson and Kell 2008; Rubio and Daniel 2008), organic anion/cations (*SLC22*) (Koepsell et al. 2007; Ciariboli 2008), and organic cations (*SLC47*) (Tanihara et al. 2007; Moriyama et al. 2008; Matsushima et al. 2009).

The *SLC25* gene encodes mitochondrial carriers (MCs), which are membrane-integrated proteins that localize to the inner membranes of mitochondria and catalyze the translocation of solutes across the membranes (Plamieri, 2004). The MCs provide a critical link between the mitochondria and the cytosol by facilitating the flux of solutes through the permeable barrier of the inner mitochondrial membrane. The substrates transported by the MCs range from the smallest H+ to the largest ATP molecule, implying that they have a broad array of functions in diverse metabolic processes. Defects in MC genes lead to several diseases such as type II citrullinaemia (SLC25A13; OMIM 215700), hyperornithine-hyperammone-homocitrulline-mia (HHH) syndrome (SLC25A15; OMIM 238970), Stanley syndrome (SLC25A20; OMIM 212138), Amish microcephaly (SLC25A20; OMIM 607196), and autosomal dominant progressive external ophthalmoplegia (adPEO) (SLC25A4; OMIM 157640). The complete amino acid sequence of the ATP/ADP carrier was identified in beef heart mitochondria (Aquila et al. 1982; Aquila et al. 1985).

Post-genomic era studies have enabled us to identify many more mitochondria carrier families (MCFs) simultaneously without laborious cloning or purification procedures. Although much is known about the characteristics and functions of MCFs in human and plants, their biological roles in fish remain unknown. In our studies, we cloned the *CcSLC25a5* (*Cyprinus Carpio SLC25a5*) gene using Genefishing kits from the skins of the mirror carp, which has interspersed scales, and the Jianli, that has full scales. The expression pattern of SLC25a5 during different developmental stages was determined by whole-mount *in situ* hybridization.

## Materials and methods

### Animals

Mirror carp and Jianli (*Cyprinus carpio Linnaeus*) were cultivated at Experimental Station of the Wuxi Freshwater Center, Jiangsu, China. The mirror carp was derived by domesticating the common carp and selecting for a scale-reduced mutation *fgfr1a* (Rohner et al. 2009). The skin tissues from mirror carp and Jianli were harvested with forceps and immediately homogenized in 1 ml Trizol (Invitrogen).

### First-strand cDNA synthesis

Total RNA extracted from the skin tissues using Trizol reagent (Invitrogen) was used to synthesis the first-strand cDNA. Subsequent reverse transcription was performed according to the manufacture’s protocol (Seegene, Seoul, South Korea). The final reaction volume was 20 uL and contained: 3 ug of purified total RNA, 4 uL of 5× reaction buffer, 5 uL of dNTPs (2 mM each), 2 uL of 10 uM dT-ACP1 (5′-GTCTACCAGGCATTCGCTTCATXXXXXGCCATCGACC-3′), 0.5 uL RNase inhibitor (40 U/uL; Invitrogen, USA), and 1 uL of reverse transcriptase (200 U/uL, Invitrogen). First-strand cDNAs were diluted using 80 uL of DNase-free water for GenefishingTM PCR, and stored at -20°C.

### ACP (Annealing Control Primer)-based Genefishing PCR

DEGs (Differential Expressed Genes) were screened by ACP-based PCR methodology using the Genefishing DEG Kits (Seegene). Briefly, second-strand cDNA was synthesized at 50°C during in the first-stage PCR reaction. The final reaction was conducted in a 20 uL volume containing: 3–5 uL of diluted first-strand cDNA, 1 uL of dT-ACP2 (10 uM), 1 uL of 10 uM arbitrary ACP (Hwang et al. 2005), and 10 uL of 2× Master Mix (Seegene). The PCR protocol for second-strand synthesis was: one cycle at 94°C for 5 minutes, followed by 50°C for 3 minutes, and 72°C for 1 minute. Once the second-strand DNA synthesis was completed, a second-stage PCR amplification protocol was conducted that consisted of: 40 cycles of 94°C for 40 seconds, 65°C for 40 seconds, and a 5 minute final extension at 72°C. The amplified PCR products were separated in a 2% agarose gel and stained with ethidium bromide.

### Cloning and sequencing

PCR bands indicating genes with differential expression were extracted from the gel using a DNA extraction kit (Zomanbio, China). The bands were directly cloned into a pEASY-T vector (Trans, China) according to the manufacturer’s instructions. The cloned plasmids were sequenced.

### Whole-mount in situ hybridization

RNA probes were prepared from a 206 bp CDS (Coding Sequence) region of the gene *SLC25a5* in common carp and labeled with digoxigenin-UTP using T3 or T7 RNA polymerase (T3 for production of the antisense probe, T7 for the sense probe). The embryonic and developmental stages of the embryos used for whole-mount *in situ* hybridization were assessed using haf (hours after fertilization) and various morphological criteria (Kane and Kimmel 1993) as described by Westerfield (1993). The RNA probes were hybridized to the tissue overnight at 65°C. The embryos and juvenile fish from each developmental stage were imaged using an Olympus BH-2 microscope (Olympus Optical, Tokyo, Japan). The primers used to create the probes were: Forward: 5′-TGGGTAACTGCTTGGTGAAGATCTCC-3′, and Reverse: 5′-ACCAGCAACAGCAGTCACAGTCTGA-3′.

## Results

### The mirror carp and Jianli have differential gene expression in skin tissues

**Figure 1:**
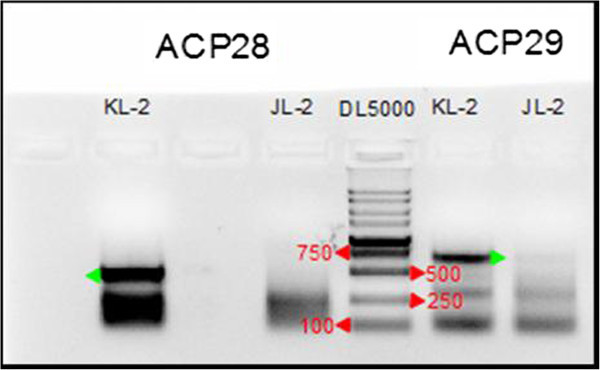
**Differential gene expression detected in the Genefishing assay using primers ACP28 and ACP29.** The PCR products from the genefishing assay were separated by agarose gel electrophoresis. Gene products that are differentially expressed in the mirror carp (KL-2) and common carp (JL-2) are shown by green arrows. DL5000 indicate molecular markers.

To identify the differentially expressed genes that are associated with development of the skin and appendages, skin samples from mirror carp and Jianli were assayed by Genefishing. The Genefishing assay used an anchored primer in combination with 20 arbitrary primers (Arbitrary ACP, Annealing Control Primer) (Table 1). We obtained seven DEGs from all ACP primers (Table 2), among these DEGs, two PCR products from ACP28 and ACP29 primers were identified that had significantly different expression levels (Figure 1). The differentially expressed bands were subcloned into pEasy-T3 vector and sequenced. The sequences were compared using the blast program in the NCBI sequence database (http://wei.sohu.com/20121011/n354628269.shtml). The blast results showed that the differentially expressed genes identified using the primers ACP28 and ACP29 were homologues of the zebrafish *SLC25a5* (So*l*ute *carrier*) and *TPT1* (*T*umor *p*rotein, translationaly-controlled1) genes, respectively. We have named them *CcSLC25a5* (The *Common carp SLC25a5*) and *CcTPT1* (The *Common carp TPT1*).

**Table 1:** **Primers used in genefishing for amplifying the differential expressed genes in skin of jianli and mirror carp**

Primers	Primer sequence
ACP1	GTCTACCAGGCATTCGCTTCATXXXXXGCCATCGACC
ACP2	GTCTACCAGGCATTCGCTTCATXXXXXAGGCGATGCC
ACP3	GTCTACCAGGCATTCGCTTCATXXXXXCCGGAGGATG
ACP4	GTCTACCAGGCATTCGCTTCATXXXXXGCTGCTCGCG
ACP5	GTCTACCAGGCATTCGCTTCATXXXXXAGTGCGCTCG
ACP6	GTCTACCAGGCATTCGCTTCATXXXXXGGCCACATCG
ACP7	GTCTACCAGGCATTCGCTTCATXXXXXCTGCGGATCG
ACP8	GTCTACCAGGCATTCGCTTCATXXXXXGGTCACGGAG
AC P9	GTCTACCAGGCATTCGCTTCATXXXXXGATGCCGCTG
ACP10	GTCTACCAGGCATTCGCTTCATXXXXXTGGTCGTGCC
ACP11	GTCTACCAGGCATTCGCTTCATXXXXXCTGCAGGACC
ACP12	GTCTACCAGGCATTCGCTTCATXXXXXACCGTGGACG
ACP13	GTCTACCAGGCATTCGCTTCATXXXXXGCTTCACCGC
ACP14	GTCTACCAGGCATTCGCTTCATXXXXXGCAAGTCGGC
ACP15	GTCTACCAGGCATTCGCTTCATXXXXXCCACCGTGTG
ACP16	GTCTACCAGGCATTCGCTTCATXXXXXGTCGACGGTG
ACP17	GTCTACCAGGCATTCGCTTCATXXXXXCAAGCCCACG
ACPI18	GTCTACCAGGCATTCGCTTCATXXXXXCGGAGCATCC
ACP19	GTCTACCAGGCATTCGCTTCATXXXXXCTCTGCGAGC
ACP2D	GTCTACCAGGCATTCGCTTCATXXXXXGACGTTGGCG

**Table 2:** **Seven candidate GOIS (gene of interest) by genefishing**

GOI number	Protein
1	Solute carrier family 25 alpha, member 5 (slc25a5)
2	Hairless protein
3	Cyprinus carpio translationally-controlled tumor protein mRNA, complete cds
4	Danio rerio myosin heavy chain, fast skeletal muscle-like, 85 bp (89%)
5	Actin, alpha, cardiac muscle 1 b [Danio rerio], 367/389 (94%)
6	Dictyostelium discoideum AX4 hypothetical protein, 84/87 (97%)
7	Fibroblast growth factor 4

### *CcSLC25a5* gene structure and its isoforms

**Figure 2:**
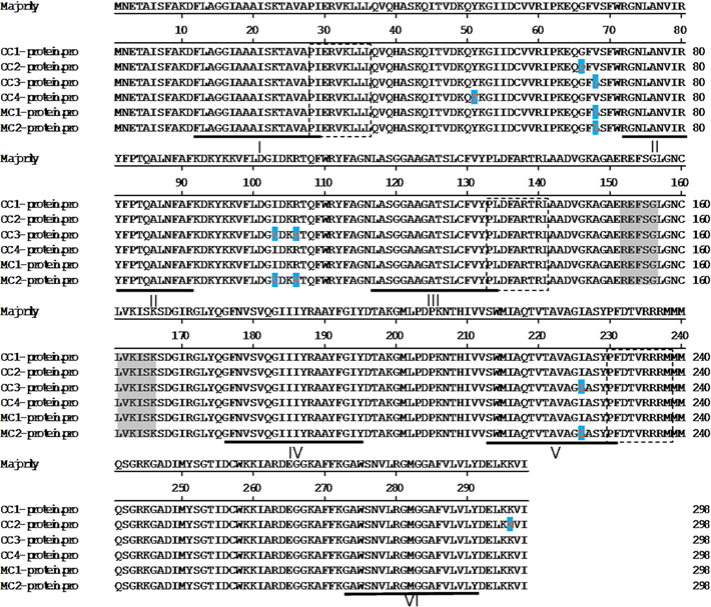
**Comparing of the amino acid sequences deduced from six carp** ***ANT2*** **transcripts.** Dotted lines under the sequences indicate the positions of six putative transmembrane helices (Klingenberg 1989) labeled segments I-VI. Three repeats of the mitochondrial carrier protein signature (Bof et al. 1999) are shown by dotted boxes. A substrate binding site is highlighted with shaded boxes. Amino acids that differ between the transcripts are indicated in blue.

One of the identified genes, *CcSLC25a5,* was selected for further analysis. We designed the primers located in the conserved region of the gene using the partial reference sequence of *CcSLC25a5* isoform 1. Nine different isoforms of the *CcSLC25a5* gene were identified in the common carp and Jianli. The genes encode six different SLC25a5 protein isoforms due to codon degeneracy. Four isoforms were found in the common carp and two isoforms were found in the mirror carp (Figure 2). The full-length *CcSLC* cDNA consists of 1697 nucleotides with an 897 base pair open reading frame (ORF). The ORF encodes a peptide of 298 amino acids with a molecular mass of 31.5 kDa and a theoretical isoelectric point of 7.49. There are seven amino acid positions that are different between the isoforms (Figure 2). SLC25a5 has six putative transmembrane domains, which are composed of three repeats of mitochondrial carrier protein features by searching the Pfam database (Finn et al. 2010). SLC25a5 also contains one substrate binding site (Figure 2) and encodes an adenine nucleotide translocator (ANT2). ANT2 (also named as SLC25a5) is the most abundant mitochondrial protein (Itoi et al. 2005).

**Figure 3:**
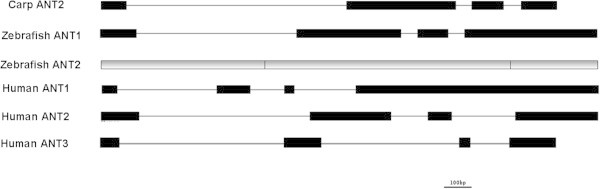
**The gene structure of the carp, zebrafish, and human** ***ANT*** **genes.** Coding regions in the exons are represented by black boxes. Introns are represented by solid lines.

Using the *CcSLC25a5* mRNA sequence as a query, we searched our assembled genome database of ***Cyprinus carpio*** (not published), two copies of *CcSLC25a5* exist on one scaffold (scaffold ID: 000000476), they are adjacent to each other and have the converse gene directions. By comparing the genomic and transcript sequences, the *CcSLC25a5* has four exons and three introns (Figure 3). We then compared the carp gene to known zebrafish and human isoforms. Zebrafish have *ANT1* (*SLC25a4*) and *ANT2* (*SLC25a5*) genes, while humans have three *ANT* genes: *ANT1*, *ANT2,* and *ANT3*. Similar to the carp, the zebrafish *ANT1* and the human *ANT1*, *ANT2*, and *ANT3* genes contain four exons (Figure 3). The exception is the zebrafish *ANT2* gene which contains three exons (Figure 3).

### Phylogenetic analysis of cyprinid fishes and mammalian SLC25a5 isoforms

**Figure 4:**
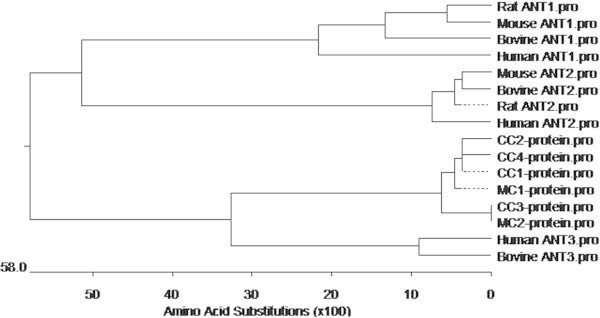
**A phylogenetic tree of carp and mammalian ANT isoforms constructed using the neighbor-joining methodology.** The evolutionary distance between amino acid substitutions was estimated using Kimura’s two parameter-method (Kimura 1980). The scale along the bottom axis shows the evolutionary distance of amino acid substitutions per site.

To analyze the phylogenetic relationship of ANTs in cyprinid fishes, a phylogenetic tree was constructed using neighbor-joining from deduced amino acid sequences (Figure 4). The six carp isoforms were clustered into one branch. They are more closely related to human and bovine ANT3, and more distantly to mammalian ANT1s (rat, mouse, bovine, human) and ANT2s (mouse, bovine, rat, human). The deduced amino acid sequence of the CcSLC25a5 protein shares a high degree of homology with other known vertebrate SLCs (82% identity with mouse, bovine, rat, human).

### Tissue expression of the *CcSLC25a5*

**Figure 5:**
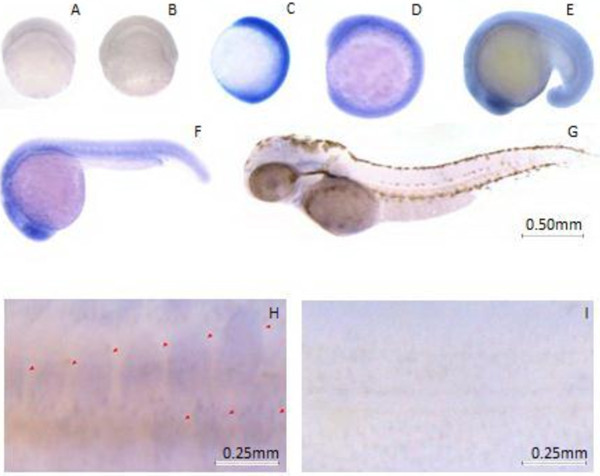
**The** ***CcSLC25a5*** **expression pattern by whole-mount in** ***situ*** **.** Panels **A-G** show the expression pattern of *CcSLC25a5* genes in different developmental stages. **(A)** 4.5 haf (1/3 epiboly), **(B)** 6 haf (shield), **(C)** 10 haf, **(D)** 12 haf, **(E)** 16 haf, **(F)** 24 haf, and **(G)** 48 haf after fertilization, respectively. **(H)** *SLC25a5* is expressed specifically in the regions where the scales were developing in the skin. **(I)** There is no hybridization signal detected using the sense probe as the negative control.

To define the timing and patterns of *CcSLC25a5* expression during early embryonic development, we performed *in situ* hybridization throughout the earlier developmental stages. The *SLC25a5* gene was expressed weakly in embryos from 4.5 (1/3 epiboly stage, Figure 5A) to 6 (shield stage, Figure 5B) hpf (hours post fertilization). However, a strong expression signal appeared at the 70% epiboly stage (10 hpf, Figure 5C). The gene expression signal was concentrated in the embryo and undetectable in the yolk. *SLC25a5* was continuously expressed beginning 10 hpf (6-somites stage) to 16 hpf (14-somites stage) (Figure 5D-F) and continuing until 48 hpf (long-pec stage, Figure 5G) when the signal gradually weakened. Interestingly, *CcSLC25a5* was specifically expressed in the scale placods as the scale pattern was formed (Figure 5H).

## Discussion

Scales of the teleost fish are important skin integumentary appendages distributed over the body surface in defined patterns. Natural variation in scale patterns exists among the common carp species. Mirror carp and Jianli are two carp varieties that have distinct scale patterns as a result of breeding selection. Scale initiation and morphogenesis are very complicated biological processes. The functional analysis of *FGFR1* by reverse genetics showed that the mutation of this gene can lead to reduced scales and abnormal fins (Rohner et al. 2009). The scale is one of the important agricultural traits for fishes and play important roles in physiology, defense, and adaptation to new environments (Sire et al. 1997). Some aquatic biologists are interested in scale development and launched some works using the reverse genetics. However, a complete knowledge of scale development is limited due to the longer sexual maturation period and the larger genome size for many species in fishes. Therefore, the molecular mechanisms underlying the scale initiation and pattern formation remain unknown. Using Genefishing, we were able to identify seven genes that were differentially expressed in Jianli and mirror carp skin tissues (Table 2). These genes may contribute to the morphogenesis of the integumentary appendages, especially the scales or fins, the functional analysis of these seven genes will be performed in future studies.

ANTs are the most abundant mitochondrial proteins and mediate the exchange of ADP and ATP across the mitochondrial membrane. They link ATP production in mitochondria to its functional utilization for energy requirements outside mitochondria (Klingenberg 1981 and 1989). Humans are known to have three *ANT* genes. *ANT1* is predominantly expressed in the heart and skeletal muscle and *ANT2* is expressed in kidney and liver. *ANT3* is expressed ubiquitously but is highest in the kidney (Stepien et al. 1992). The tissue distributions of different ANT isoforms seem to reflect their functional differences and associate with tissue-specific energy metabolism (Stepien et al. 1992).

Given their tissue specific roles in humans, it was of interest to examine expression profiles of ANT isoforms in various tissues in the carp. To date, there are no reports about *ANT* expression in fish scales. Herein, we report that ANT2 is specifically expressed in the scale placods. This result indicates that ANT2 may have an important role in scale development and morphogenesis. It has been suggested that fish ANTs have an important role in energy production, which is associated with temperature adaptation in fish. Guderley and Johnston (1996) reported that the uptake of ADP in isolated mitochondria from cold-acclimated sculpin was higher than that of their warm-acclimated counterparts. *CcSLC25a5* possibly involved in the energy production in scale development for fishes, so it should be expressed in the process of scale development. Additional studies will provide new insights into the regulation of energy metabolism during scale development in fish.

We next examined the expression profiles of *CcSLC25a5* in various tissues of the Jianli during development. *CcSLC25a5* was weakly expressed in early stages of embryo development (4.5 to 6 hpf, Figure 5A-B). However, strong expression signals were detected in embryos from 10 to 24 hpf (Figure 5C-F). These results imply that *CcSLC25a5* has roles in embryo and scale development.

## References

[CR1] Almen MS, Nordstrom KJ, Fredriksson R, Schioth HB (2009). Mapping the human membrane proteome: a majority of the human membrane proteins can be classified according to function and evolutionary origin. BioMed Central Biology.

[CR2] Aquila H, Misra D, Eulitz M, Klingenberg M (1982). Complete amino acid sequence of the ADP/ATP carrier from beef heart mitochondria. Hoppe-Seyler’s Z Physiol Chem.

[CR3] Aquila H, Link TA, Klingenberg M (1985). The uncoupling protein from brown fat mitochondria is related to the mitochondrial ADP/ATP carrier. Analysis of sequence homologies and of folding of the protein in the membrane. EMBO J.

[CR4] Bof M, Brandolin G, Satre M, Klein G (1999). The mitochondrial adenine nucleotide translocator from *Dictyostelium discoideum.* Functional characterization and DNA sequencing. European Journal of Biochemistry.

[CR5] Brandsch M, Knutter I, Bosse-Doenecke E (2008). Pharmaceutical and pharmacological importance of peptide transporters. J Pharm Pharmacol.

[CR6] Ciariboli G (2008). Organic cation transporters. Xenobiotica.

[CR7] Dobson PD, Kell DB (2008). Carrier-mediated cellular uptake of pharmaceutical drugs: an exception or the rule?. Nat Rev Drug Discov.

[CR8] Finn RD, Mistry J, Coggill P, Heger A, Pollington JE, Gavin OL, Gunasekaran P, Ceric G, Forslund K, Holm L, Sonnhammer EL, Eddy SR, Bateman A (2010). The Pfam protein families database. Nucleic Acids Res.

[CR9] Fredriksson R, Nordström KJV, Stephansson O, Hägglund MGA, Schiöth HB (2008). The solute carrier (SLC) complement of the human genome: Phylogenetic classification reveals four major families. FEBS Lett.

[CR10] Guderley H, Johnston LA (1996). Plasticity of fish muscle mitochondria with thermal acclimation. J Exp Biol.

[CR11] Hediger MA, Michael F, Romero MF, Peng JB, Rolfs A, Takanaka H, Bruford EA (2004). The ABCs of solute carriers: physiological, pathological and therapeutic implications of human membrane transport proteins. Pflügers Archive European Journal of Physiology.

[CR12] Hwang DY, Cho JS, Oh JH, Shim SB, Jee SW, Lee SH, Seo SJ, Lee SK, Lee SH, Kim YK (2005). Differentially Expressed Genes in Transgenic Mice Carrying Human Mutant Presenilin-2 (N141I): Correlation of Selenoprotein M with Alzheimer’s Disease. Neurochem Res.

[CR13] Itoi S, Misaki R, Hirayama M, Nakaniwa M, Liang CS, Kondo H, Watabe S (2005). Identification of three isofoms for mitochondrial adenine nucleotide translocator in the pufferfish Takifugu rubripes. Mitochondrion.

[CR14] Kane DA, Kimmel CB (1993). The zebrafish midblastula transition. Development.

[CR15] Kimura M (1980). A simple method for estimating evolutionary rates of base substitutions through comparative studies of nucleotide sequences. J Mol Evol.

[CR16] Klingenberg M (1981). Membrane protein oligomeric structure and transport function. Nature.

[CR17] Klingenberg M (1989). Molecular aspects of the adenine nucleotide carrier from mitochondria. Arch Biochem Biophys.

[CR18] Koepsell H, Lips K, Volk C (2007). Polyspecific organic cation transporters: structure, function, physiological roles, and biopharmaceutical implications. Pharm Res.

[CR19] Lagerstrom MC, Schioth HB (2008). Structural diversity of G protein-coupled receptors and significance for drug discovery. Nat Rev Drug Discov.

[CR20] Matsushima S, Maeda K, Inoue K, Ohta KY, Yuasa H, Kondo T, Nakayama H, Horita S, Kusuhara H, Sugiyama Y (2009). The inhibition of human multidrug and toxin extrusion 1 is involved in the drug-drug interaction caused by cimetidine. Drug Metabolism Disposition.

[CR21] Moriyama Y, Hiasa M, Matsumoto T, Omote H (2008). Multidrug and toxic compound extrusion (MATE)-type proteins as anchor transporters for the excretion of metabolic waste products and xenobiotics. Xenobiotica.

[CR22] Plamieri F (2004). The mitochondrial transporter family (SLC25): physiological and pathological implications. Pflügers Archive European Journal of Physiology.

[CR23] Ren Q, Chen K, Paulsen IT (2007). Transporter DB: A comprehensive database resource for cytoplasmic membrane transport systems and outer membrane channels. Nucleic Acids Res.

[CR24] Rohner N, Bercsényi M, Orbán L, Kolancayk ME, Linke D, Brand M, Nüsslein-Volhard C, Harris MP (2009). Duplication of fgfr1 Permits Fgf Signaling to Serve as a Target for Selection during Domestication. Curr Biol.

[CR25] Rubio AI, Daniel H (2008). Peptide transporters and their roles in physiological processes and drug disposition. Xenobiotica.

[CR26] Russel FG, Masereeuw R, Van ARA (2002). Molecular aspects of renal anionic drug transport. Annu Rev Physiol.

[CR27] Sire JY, Allizard F, Babiar O, Bourguignon J, Quilhac A (1997). Scale development in zebrafish (Danio rerio). J Anat.

[CR28] Sreedharan S, Stephansson O, Schiöth HB, Fredriksson R (2011). Long evolutionary conservation and considerable tissue specificity of several atypical solute transporters. Gene.

[CR29] Stepien G, Torroni A, Chung AB, Hodge JA, Wallace DC (1992). Differential expression of adenine nucleotide translocator isoforms in mammalian tissues and during muscle cell differentiation. J Biol Chem.

[CR30] Tanihara Y, Masuda S, Sato T, Katsura T, Ogawa O, Inui K (2007). Substrate specificity of MATE1 and MATE2-K human multidrug and toxin extrusions/H(+)-organic cation antiporters. Biochemistry Pharmacology.

[CR31] Westerfield M (1993). The zebrafish book: a guide for the laboratory use of zebrafish (Brachydanio rerio).

